# A metabolite-dependent mechanism by which *Bifidobacterium animalis* subsp. *lactis* promotes *Bacteroides* colonization

**DOI:** 10.1080/19490976.2026.2696647

**Published:** 2026-07-07

**Authors:** Khashayar Shahin, Liang Wang, Zihan He, Bomin Lv, Arya Van Alin, Richard Lo-Man, Hao Wu, Philippe Sansonetti, Jean-Marc Collard

**Affiliations:** a State Key Laboratory of Genetic and Development of Complex Phenotypes, Fudan Microbiome Center, School of Life Sciences, and Human Phenome Institute, Fudan University, Shanghai, People's Republic of China; b Laboratory Medicine, Guangdong Provincial People's Hospital (Guangdong Academy of Medical Sciences), Southern Medical University, Guangzhou, Guangdong Province, People's Republic of China; c Division of Microbiology and Immunology, School of Biomedical Sciences, University of Western Australia, Perth, WA, Australia; d School of Agriculture and Food Sustainability, University of Queensland, Brisbane, Australia; e Shanghai Institute of Immunity and Infection, Chinese Academy of Sciences, Shanghai, People's Republic of China; f Curtin Medical School, Curtin University, Perth, WA, Australia; g Microbial Ecology Laboratory, School of Science, Edith Cowan University, Perth, WA, Australia; h Department of Biology and Interdisciplinary Nanoscience Center (iNANO), Aarhus University, Aarhus, Denmark; i Center for Obesity and Hernia Surgery, Department of General Surgery, Huashan Hospital, Fudan University, Shanghai, People's Republic of China; j Institut Pasteur of Shanghai, Chinese Academy of Sciences (IPS-CAS), 320 Yueyang Road, Shanghai, People's Republic of China

**Keywords:** Gut microbiome, *Bifidobacterium*, *Bacteroides*, early colonization, metabolites

## Abstract

Prokaryote-prokaryote symbiotic relationships influence interactions within microbial communities, affecting colonization, survival, and organization. Unlike competition, consortium species facilitate growth via metabolite cross-feeding. This study explored interactions between two early human gut colonizers: partially aerotolerant *Bifidobacterium* spp. and strict anaerobic *Bacteroides* spp., using omics techniques. Promotion of *Bacteroides* spp. growth by *Bifidobacterium animalis* subsp. *lactis* was demonstrated through co-culture experiments in anaerobic conditions. Metabolomic analysis revealed over 150 unique metabolites present in *B. animalis* subsp. *lactis* supernatants are absent in other *Bifidobacterium* species, including 3-hydroxycapric acid, D-alanyl-D-alanine, 2-isopropylmalic acid, and D-glucose 2-phosphate. These compounds served as nutritional substrates, including carbon and nitrogen sources, significantly enhancing *Bacteroides* spp. growth. In murine models, early colonization by *B. animalis* subsp. *lactis* consolidated *Bacteroides fragilis* colonization (1.7 × 10^4^ to 9.7 × 10^6^ copy number/g fecal sample) by providing these metabolites as a niche. These findings highlight *B. animalis* subsp. *lactis* plays a critical role in gut colonization of *Bacteroides* spp. via its exclusive metabolic profile, offering insights into partitioned metabolic activity within gut communities and emphasizing the importance of specific metabolites in early microbial establishment.

## 1. Introduction

The first days and weeks of human life represent a series of critical windows of opportunity for shaping the development of the gastrointestinal tract, immune system, and future adult microbiome.^1^ Several studies have shown that facultative anaerobes are the first settlers in the primitive gut, pointing toward a relatively aerobic intestinal environment.^2^ ^,^ ^3^ This suggests that the pioneer microbiota core is comprised predominantly of facultative anaerobes such as *Pseudomonadota* and *Bacillota*, but rapidly transitions to a *Bifidobacterium*-dominated microbiota within a few days after birth,^4^ ^,^ ^5^ especially in breast-fed infants. In contrast, obligate anaerobe families or genera belonging to *Bacillota* (*Clostridiaceae*, *Lachnospiraceae*, *Ruminococcaceae*, *Veillonellaceae*), *Bacteroidota* (*Bacteroidaceae*, *Porphyromonadaceae*, *Prevotellaceae*), and *Actinomycetota* (*Propionibacteriaceae*) become predominant later on during infancy and early childhood. By combining available metagenomes from nine distinct studies comprising over 1944 samples (441 infants from 415 families), Podlesny and Frick reported that vaginally-delivered infants started with a large proportion of strict anaerobe species, e.g., *Bacteroides*, which constituted more than 50% of the total microbiota by day four.^6^ In the first six months of life, the differences between vaginally-delivered infants and infants born by C-section included increased relative abundances of the *Bifidobacteria* (*B. longum* and *B. bifidum)*, the *Bacteroidales* (*B. dorei* and *B. fragilis*), *Parabacteroides distasonis,* and the *Enterobacteriaceae* (*Escherichia coli)*.

We were interested in bacterial interactions within the gut microbiota, particularly those that shape colonization and the formation of bacterial communities, which contribute to the maturation, resilience, and stability of the infant gut ecosystem. Previous studies have explored co-cultures involving strains from two *Bifidobacterium* species and two *Bacteroides* species, using purified exopolysaccharides (EPS) from *Bifidobacteria*, inulin, or glucose as carbon sources. The results have revealed distinct metabolic and compositional shifts depending on the specific strain pairings and substrates, highlighting that microbial interactions in the gut are highly strain-dependent. Such findings caution against oversimplified generalizations across broader taxonomic levels and emphasize the nuanced dynamics that govern microbial cross-feeding and colonization potential.^7^ Therefore, we hypothesized that pre-colonizing *Bifidobacterium* spp. produces certain metabolites that support colonization of *Bacteroides* spp. by providing nutritional advantages. In the present study, in vivo and in vitro experiments were performed to understand the potential effects of four *Bifidobacterium* species on *the* growth and colonizing rates of four *Bacteroides* species. The findings underscore a symbiotic relationship between *Bifidobacterium animalis* subsp. *lactis* and *Bacteroides fragilis*, demonstrating produced metabolites by *B. animalis* subsp. *lactis* significantly enhance the colonization and stabilization of *B. fragilis* both in vitro and in murine models.

## 2. Results

### 2.1. Synergistic growth between *Bifidobacterium* spp. and *Bacteroides* spp. in co-cultures

BHI agar was used for the co-cross-streak plate assay. Four of the most abundant *Bifidobacterium* spp. (*B. animalis* subsp. *lactis, B. bifidum, B. breve,* and *B. longum*) and four most abundant species of *Bacteroides* spp. (*B. dorei, B. fragilis, B. ovatus,* and *B. thetaiotaomicron*) were initially screened using the perpendicular streak method (Figure S1A). *Bifidobacterium* spp. grew on BHI agar in strictly anaerobic conditions after approximately 36 h, while no *Bacteroides* spp. colonies were observed until the end of the incubation period (72 h) (Figure S1B). In contrast, when *B. animalis* subsp. *lactis* was grown in combination with *B. dorei, B. fragilis, B. ovatus,* and *B. thetaiotaomicron*, all four *Bacteroides* species showed substantial growth, and typical colonies were visible on plates (Figure S1C). On the other hand, *B. bifidum* slightly promoted the growth of only *B. ovatus* (Figure S1C), while *B. breve* and *B. longum* did not support *Bacteroides* spp. growth used in this study (Figure S1D). It is worth noting that the presence of *Bacteroides* spp. conversely did not improve the growth of *B. animalis* subsp. *lactis*.

### 2.2. Growth of *Bacteroides* spp. depends on soluble components produced by *Bifidobacterium. animalis* subsp. *lactis*

**Figure 1.**
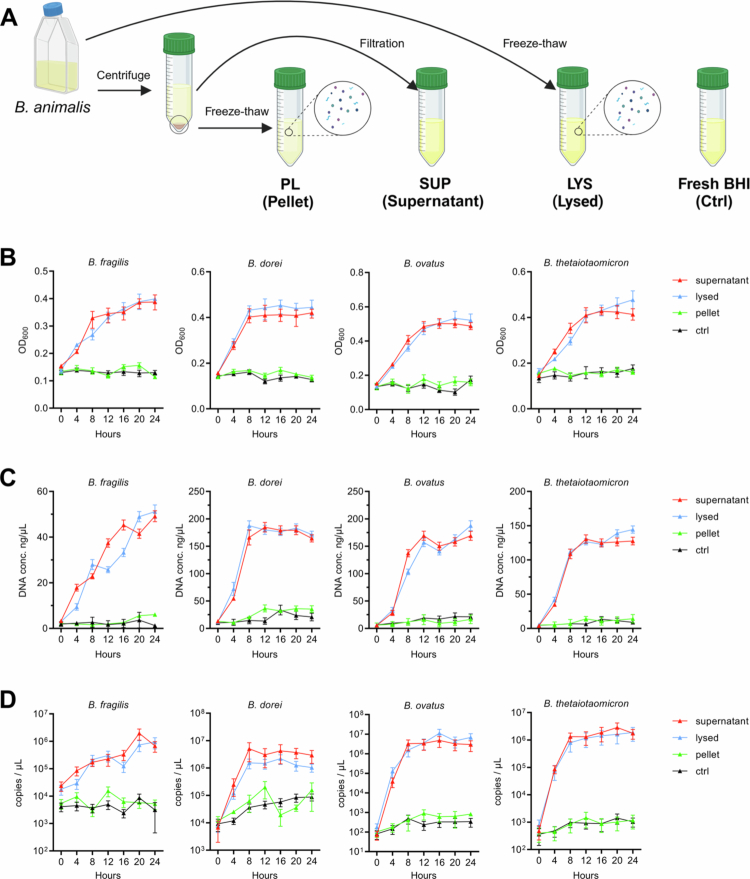
Evaluation of *Bacteroides* spp. growth dependency on compounds derived from *Bifidobacterium animalis* subsp. *lactis*. (A) Experimental setup to analyze the effect of *B. animalis* subsp. *lactis*-derived compounds on *Bacteroides* spp. growth. B) growth (OD_600_), C) total biomass (DNA concentration), and D) copy number of individual *Bacteroides* spp. at the endpoint (24 h) in minimal media (MM) broth supplemented with *B. animalis* lysed cells in broth culture (LYS), lysed pellet only (PL), and supernatant only (SUP). Data points represent mean ± SEM. Growth of *Bacteroides fragilis* in the presence of untreated and reagent-treated *B. animalis* culture supernatants. OD₆_00_ was monitored over 24 hours to assess the impact of various treatments on the growth-promoting activity of *Bifidobacterium* supernatant (SUP or LYS solutions without *Bacteroides* inoculation as the blank for background correction). Error bars indicate standard deviation from biological replicates (*n* = 3). Exact *P* values and test statistics are reported in Table S1.

To gain insight into the role of *B. animalis* subsp. *lactis* in promoting the growth of *Bacteroides* spp., minimal medium (MM) was supplemented with different suspensions (LYS, PL, SUP) of the *B. animalis* subsp. *lactis* culture under strictly anaerobic conditions (Figure 1A). *Bacteroides* spp. did not form colonies on the MM agar plates supplemented with the *B. animalis* lysed pellet (PL) (Figure S2). However, colony formation was evident on the MM agar plates supplemented with the supernatant (SUP) from the *B. animalis* liquid culture or the lysed bacteria (LYS). There was no significant difference in *Bacteroides* spp. growth in terms of OD_600_, biomass (DNA concentration), and cell number (copy number) between SUP and LYS groups; but both groups showed significantly higher growth compared to the MM and PL conditions (Figure 1B). These results suggest that *B. animalis* subsp. *lactis* cells release soluble compounds into the medium that could enhance*Bacteroides* spp. growth. While these compounds appear to play a key role, the growth-promoting effect may also reflect a combination of mechanisms inherent to the LYS condition. In contrast, overall *Bacteroides* cell counts did not significantly differ between LYS and SUP supplementation, nor between PL and the control, suggesting the extracellular nature of the growth-promoting compounds (Figure 1B–D). Collectively, these findings highlight the necessity of supportive nutrition for *Bacteroides* spp. in the *Bifidobacterium-Bacteroides* interaction. *Bacteroides* spp. derived a unilateral growth advantage under limited culture conditions in the presence of compounds produced by *B. animalis* subsp. *lactis*.

### 2.3. Physicochemical and biochemical treatments of *B. animalis* subsp. *lactis* supernatant reveals distinct molecular contributors to *B. fragilis* growth stimulation

To identify the molecular determinants responsible for the growth-promoting activity of *B. animalis* subsp. *lactis* supernatant (SUP), we subjected the SUP to a series of targeted physicochemical and biochemical treatments and assessed their impact on *B. fragilis* growth over 24 h under anaerobic conditions (Figure S3). Redox adjustment of the SUP to the physiological anaerobic range (−350 to −400 mV) resulted in a moderate change in *B. fragilis* growth, suggesting that slightly redox-sensitive metabolites contribute to the stimulatory effect. Mild heat treatment at 65 °C for 60 min preserved most of the activity, indicating that the majority of active compounds are heat-stable. In contrast, activated charcoal treatment, which removes the majority of metabolites such as hydrophobic and aromatic compounds, substantially impaired growth, pointing to the importance of nonpolar small molecules. Neutralization of the SUP to pH 7.0 slightly reduced its early growth-promoting capacity, consistent with the presence of pH-sensitive metabolites whose activity depends on the native acidic environment. Molecular weight fractionation revealed that the <3 kDa fraction retained most of the growth-enhancing effect, while the >3 kDa fraction was largely inactive, indicating that small-molecule metabolites are the primary drivers. Severe heat treatment at 95 °C for 10 min markedly diminished growth, confirming that a subset of heat-labile components is also required. Proteinase K digestion further reduced the stimulatory effect, implicating peptide-based and bioactive proteins (Figure S3). Together, these results demonstrate that the growth-enhancing activity of *B. animalis* subsp. *lactis* SUP arises from a combination of redox-sensitive, pH-dependent, hydrophobic, small-molecule, and heat-labile peptide/protein components. The differential impact of each treatment provides mechanistic insight into the nature of the active compounds and supports a multifactorial model of cross-species metabolic facilitation.

### 2.4. Exclusive metabolic profile of *Bifidobacterium animalis* subsp. *lactis* revealed by metabolomics

**Figure 2.**
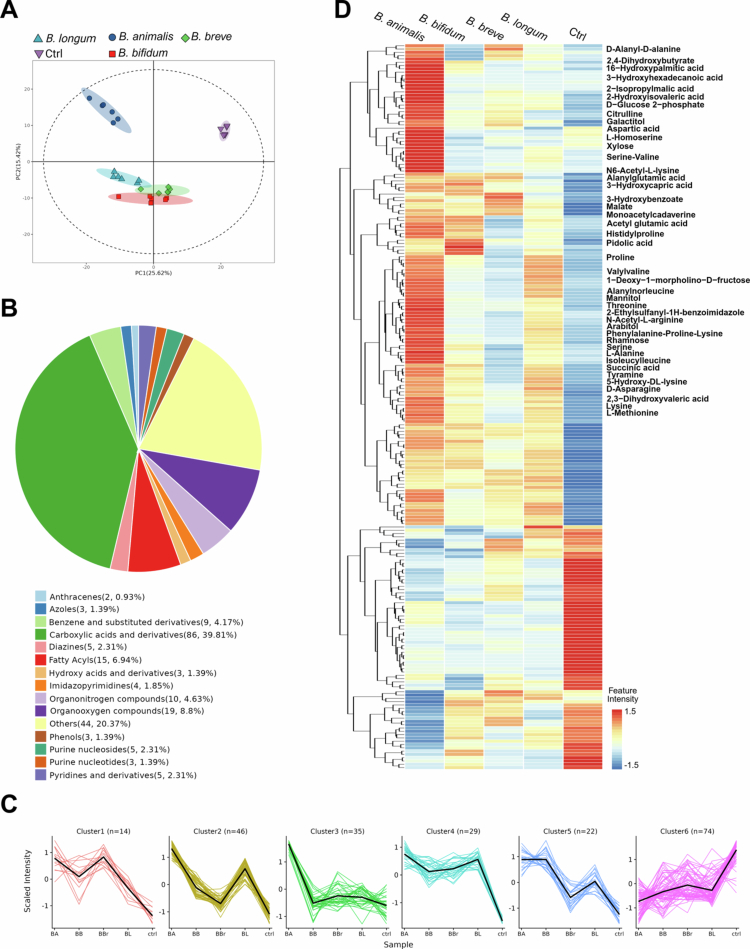
Substances produced by and involved in the metabolism of *Bifidobacterium animalis* subsp. *l* *actis*. (A) Principal component analysis (PCA) of metabolomics profiles based on PC1 and PC2 from mean intensity values of all detected metabolites. Shaded ellipses represent 95% confidence regions for each group. Statistical significance of group separation was assessed using PERMANOVA (*P* = 0.001). (B) The pie chart shows the chemical classes identified by untargeted metabolomics and their relative abundance in the *Bifidobacterium* spp. supernatants. (C) The hierarchical clustering of metabolites demonstrates distinct chemical patterns. Scaled average metabolite intensities across the *Bifidobacterium* species were hierarchically clustered with complete linkage and cut into discrete clusters with a height of 1.5, distinguishing *B. animalis* subsp. *lactis, B. bifidum, B. breve*, *B. longum,* and the control metabolites. Annotated metabolites for each cluster and their MSI confidence level are listed in Table S2. (D) Heatmap of individual key metabolites in cultures *B. animalis* subsp. *lactis, B. bifidum, B. breve*, *B. longum,* and the control. Features shown are those whose abundance significantly differed from controls (FDR-adjusted *p* < 0.1 and absolute log2 fold change > 0.75), refer to Figure S7 for fully annotated heatmap. The potentially important identified metabolites are labeled. The values shown are the average log-transformed peak heights of six replica, scaled for each feature (Table S2, Figures S4 to S7).

The aforementioned data clearly demonstrated that *B. animalis* subsp. *lactis* culture supernatant stimulated the growth of *Bacteroides* spp. Consequently, we investigated the specific components within the supernatant. *B. animalis* subsp. *lactis* and the other *Bifidobacterium* species were inoculated in BHI broth and incubated anaerobically for 24 hours. To identify key nutrients, metabolites, and metabolic pathways, the collected supernatants from *Bifidobacterium* species broth cultures were analyzed using untargeted metabolomics. This analysis identified the primary metabolites utilized or produced by *B. animalis* subsp. *lactis* and highlighted active core metabolic pathways. The metabolites were putatively identified by accurate mass and MS/MS data, matched against HMDB, MassBank, and the local self-built metabolite standard library (M&M Section). The PCA plot analysis of untargeted metabolomic profiling revealed that the metabolites found in *B. animalis* subsp. *lactis* culture media significantly differed from those found in other *Bifidobacterium* species culture media (*B. bifidum, B. breve*, and *B. longum*) (Figure 2A and Table S2). The numerous detected compounds indicated a heterogeneous mixture of unique metabolites.

Out of 625 detected compounds, 303 (48.5%) showed significantly different abundance in *B. animalis* subsp. *lactis* compared to the control (sterile medium), of which the majority (208, 64.64%), showed an increased level (Figure S4A). Moreover, remarkable differences (substantial increase) in diversity and abundance of metabolites were observed in the cases of *B. animalis* subsp. *lactis* vs. *B. bifidum* (176, 64.23%), *B. breve* (173, 61.78%) or *B. longum* (151, 61.38%) (Figure S4B–D). The produced metabolites by *B. animalis* subsp. *lactis* were mostly related to carboxylic acids (39.81%), organooxygen compounds (8.8%), and fatty acids (6.94%) (Figures 2B and S5). Differential-abundance analysis of the metabolites revealed that *Bifidobacterium* species used in the present study have different metabolite profiles (Figure 2C and Table S2). Specifically, among the metabolites, compounds related to long-chain fatty acids (LCFA), medium-chain fatty acids (MCFA), short-chain fatty acids (SCFA), sugar compounds, vitamin B-related metabolites, and a long list of amino acids demonstrated significant differences among the four species (Figure 2C and D, Figure S6 and Table S2).

**Figure 3.**
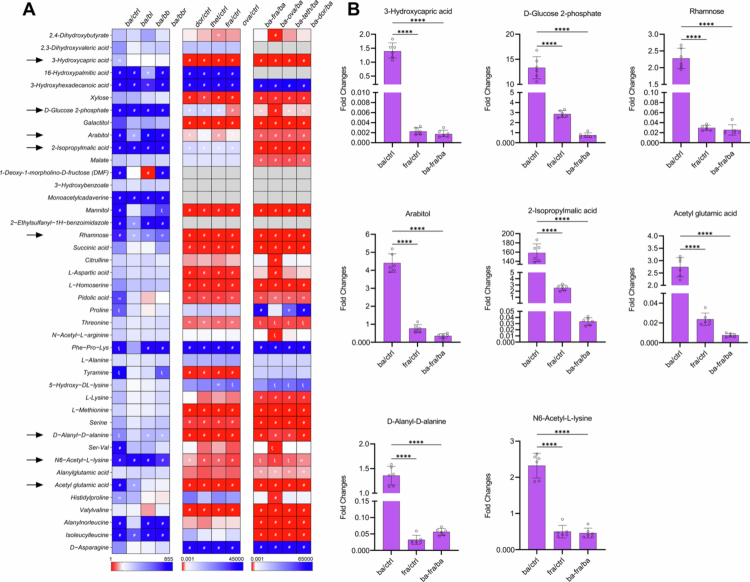
Utilization of *Bifidobacterium animalis* subsp. lactis -derived metabolites by *Bacteroides* spp. (A) Heatmap illustrates the relative abundance of 42 key metabolites significantly elevated in *B. animalis* subsp. *lactis* cultures (left panel). The middle panel shows the relative abundance of these metabolites in *Bacteroides* spp. supernatant compared to control (sterile medium). The right panel displays the relative abundance of the metabolites in *Bacteroides* cultures grown in *B. animalis* supernatant. bl, *B. longum*; bbr, *B. breve*, ba, *B. animalis*; bb. *B. bifidum*; dor, B. *dorei;* fra*, B. fragilis;* ova, *B. ovatus* and thet, *B. thetaiotaomicron.* The columns compare groups based on fold change values (*n* = 6). *Π*, *P* < 0.05; *ξ*, *P* < 0.01; #, *P* < 0.001. (B) Bar charts of the selected metabolites, marked with black arrows in panel A, represent fold changes calculated from paired comparisons based on mean metabolite abundances obtained from six biological replicates per group (All data are provided in Tables S2 and S3). These bar-charts show the fold changes of the selected metabolites in *B. animalis* subsp. *lactis* versus ctrl (ba/ctrl), *B. fragilis* versus ctrl (fra/ctrl), and *B. fragilis* culture grown in *B. animalis* subsp. *lactis* supernatant versus *B. animalis* (ba-fra/ba) groups. Statistical significance was determined using Tukey's multiple comparisons test (**P < 0.01 and ***P < 0.001).

A subset of these 42 metabolites, which were prominently detected in the supernatant of *B. animalis* subsp. *lactis* culture, could serve as potential substrates that facilitate the growth of *Bacteroides* spp., as observed in symbiotic interaction assays outlined previously (Table S3). Metabolomics analysis of the supernatant derived from cultures grown in the presence of *Bacteroides* spp. revealed that these microorganisms also produced and secreted several metabolites in high quantities, with their abundances increasing significantly compared to the control (Figure 3A, middle panel). Additionally, the observed significant decreases in the quantities of these specific metabolites in *Bacteroides* cultures grown in *B. animalis* subsp. *lactis* supernatant indicated their utilization by *Bacteroides* (Figure 3A, right panel) (Table S3).

To identify key metabolites involved in the cross-feeding interaction, compounds produced exclusively by *B. animalis* subsp. *lactis*, but not by *Bacteroides* spp., were first catalogued. From this unique set, only those metabolites whose concentrations decreased when *Bacteroides* spp. were cultured with *B. animalis* subsp. *lactis* supernatant were selected, indicating active microbial consumption. This two-step strategy enabled precise identification of metabolites that were synthesized by *B. animalis* subsp. *lactis* and utilized by *Bacteroides* spp., underscoring their specific role in metabolic cross-feeding. Notably, 3-hydroxycapric acid, arabitol, rhamnose, D-alanyl-D-alanine, N6-acetyl-L-lysine, and acetyl glutamic acid were identified as key differential metabolites. As demonstrated in Figure 3B, while *B. fragilis* exhibited the ability to produce 2-isopropylmalic acid (Fold change = 2.61 in fra/ctrl, *p* > 0.001) and D-glucose 2-phosphate (fold change = 2.91 in fra/ctrl), *B. animalis* subsp. *lactis* proved to be a more prolific producer. The relative abundance of these metabolites increased significantly to 168.08 folds (*p* > 0.001) and 14.66 (*p* > 0.001), respectively in *B. animalis* subsp. *lactis* cultures. This significant production, coupled with the significant decreases in the quantities of 2-isopropylmalic acid (Fold change = 0.033 in ba-fra/ba, *p* > 0.001) and D-glucose 2-phosphate (Fold change = 0.83 in ba-fra/ba, *p* > 0.001) in *Bacteroides* spp. cultures grown in *B. animalis* subsp. *lactis* supernatant, indicates their utilization by *Bacteroides* spp. under minimal nutritional conditions. These findings underscore the crucial role of *B. animalis* subsp. *lactis* in modulating the metabolic landscape to favor the growth of *Bacteroides* spp., highlighting a symbiotic interaction that facilitates mutualistic growth and sustenance (Figure 3B).

### 2.5. Bifidobacterium animalis subsp. *lactis* and its derived compounds promoted the colonization of mouse intestine by *Bacteroides fragilis* in vivo

**Figure 4.**
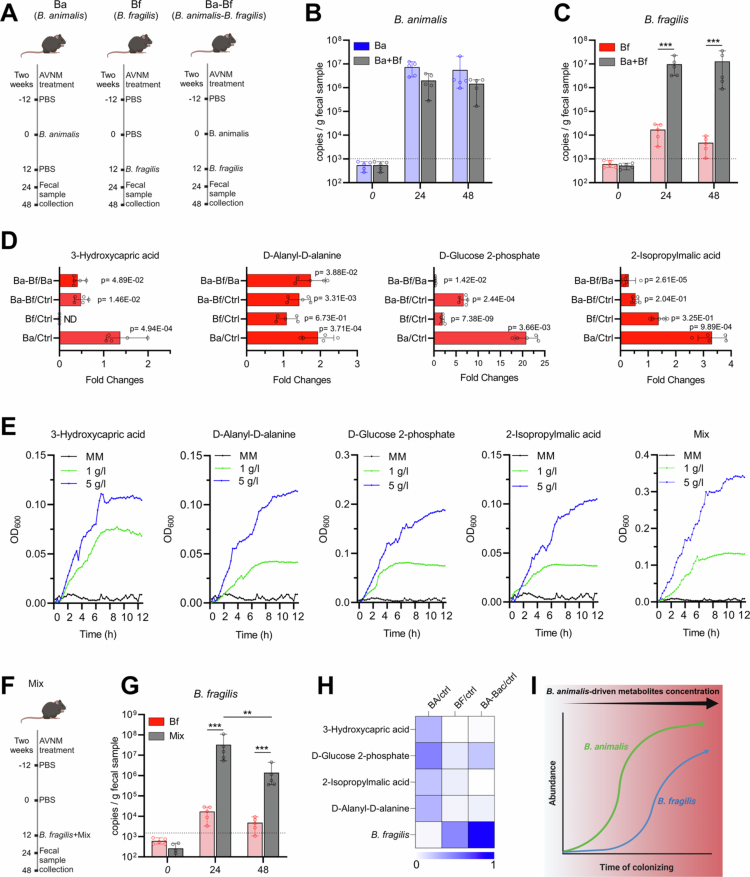
*Bifidobacterium animalis* subsp. *lactis* promotes *Bacteroides fragilis* growth in vivo through metabolite production. (A) Schematic of the experimental setup assessing the impact of *B. animalis* subsp. lactis on *B. fragilis* colonization in antibiotic-treated C57BL/6 mice (*n* = 5). Mice were treated with a broad-spectrum antibiotic cocktail to deplete the native gut microbiota, followed by administration of *B. animalis* subsp. *lactis* at ∼6 × 10^7^ CFU per mouse, following the experimental design. (B–C) Quantitative PCR analysis of fecal samples collected at day 0, 1, 2, 7 and 14 post-administration, revealing the copy number dynamics of *B. animalis* and *B. fragilis*. Both species were initially below the detection threshold (10^3^ copies/μl). Each data point represents one animal (*n* = 5/group). (D) Bars represent fold changes calculated from paired comparisons based on mean metabolite abundances obtained from five biological replicates per group. Statistical significance was determined using the original replicate measures and the calculated *p*-values are shown on each bar. (E) In vitro growth curves of *B. fragilis* cultured in mice cecum fluid supplemented with individual metabolites or a combined metabolite mixture. Growth was assessed via OD₆₀₀ over 12 hours of anaerobic incubation, across four biological replicates. (F–G) In vivo evaluation of the effect of metabolite mixture on *B. fragilis* colonization following co-administration. Bacterial copy numbers were monitored over time using qPCR. (H) Single-color heatmap showing the relative abundance of key metabolites and *B. fragilis* across the experimental groups (BA/ctrl, BF/ctrl, and BA–Bac/ctrl). Color intensity reflects normalized values, with darker shades indicating higher abundance. (I) Proposed model of nutritional interdependence between *B. animalis* and *B. fragilis*. The metabolites produced by early colonizing *B. animalis* subsp. *lactis* establish a favorable environment that supports subsequent colonization and growth of *B. fragilis*. Significance was assessed using Kruskal-Wallis tests (*P < 0.05, **P < 0.01, ***P < 0.001). Metabolite data are presented as log-transformed peak heights, averaged from five replica and scaled by feature (Table S2).

To assess whether *B. animalis* subsp. *lactis* and its derived metabolites could enhance the colonization of *B. fragilis* in the gut, we employed a murine model. Mice were used to evaluate the impact of *B. animalis* subsp. *lactis* colonization on the growth of *B. fragilis*, with particular attention to metabolic contributions (Figure 4A). Antibiotic-treated AVNM mice^8^ were successfully colonized by *B. animalis* subsp. *lactis* throughout the entire experimental period (Figure 4B). Importantly, all mice maintained relatively stable body weight throughout the 14-day study, with no signs of distress or adverse health effects observed during routine monitoring, indicating that the treatments were well tolerated (Figure S9A). In mice co-administered *B. animalis* subsp. *lactis* and *B. fragilis* (Ba-Bf), the *B. fragilis* copy number increased significantly to 9.75 × 10^6^ copies/g feces on day 1 (*p* < 0.001) and remained elevated through day 14, reaching 2.8 × 10^6^ copies/g feces (*p* < 0.001) (Figure 4C, Figure S9B and C). This increase in *B. fragilis* copy number correlated with the presence of key metabolites such as 3-hydroxycapric acid, D-alanyl-D-alanine, 2-isopropylmalic acid, and D-glucose 2-phosphate. As shown in Figure 4D (Tables S2 and S3), these metabolites were abundant in the feces of mice administered *B. animalis* compared to the control group (Ba/ctrl), whereas they were quantified in low amounts (or absent) in mice administered with *B. fragilis* alone. Additionally, in mice pre-administered with *B. animalis* subsp. *lactis* and subsequently colonized with *B. fragilis* (Ba-Bf), the levels of these metabolites significantly decreased compared to those in mice administered with *B. animalis* subsp. *lactis* alone (Ba-bac/Ba) and the control group (Ba-bac/ctrl) (Figure 4D, Figure S9B and C).

The growth curve analysis of *B. fragilis* in the presence of 3-hydroxycapric acid (5 mM), D-alanyl-D-alanine (6 mM), 2-isopropylmalic acid (1 mM), and D-glucose 2-phosphate (4 mM) demonstrated that growth promotion was dose-dependent. Moreover, when these metabolites were combined, their efficacy in promoting growth surpassed that of the individual metabolites (Figure 4E).

In vivo test was also done by feeding the mixed metabolites solution, administered via gavage, to the mice (Figure 4F). The *B. fragilis* cell copy number increased significantly, reaching 3.27 × 10^7^ copies/g feces after a day (*p* < 0.001). A modest decline was observed by day 2, with levels decreasing to 1.39 × 10^6^ copies/g feces (*p* < 0.01), followed by a further reduction to a non-significant level (1.4 10^4^ copies/g feces) by day 14. (Figure 4G). It is worth noting that the presence of *B. animalis* subsp. *lactis* could ensure a continuous supply of metabolites, maintaining the *B. fragilis* count at day 1 (9.7 × 10^6^ copy number/g fecal sample) and day 14 (2.8 × 10^6^ copy number/g fecal sample) in the gut up to the end of the experiment period (Figure 4C), whereas administration of the metabolites promotes a temporary increase in *B. fragilis* at day 1 (3.27 × 10^7^ copy number/g fecal sample) followed by a constant decline at day 14 (1.4 10^4^ copy number/g fecal sample, *p* < 0.001) (Figure 4G).

A time-dependent increase in *B. fragilis* abundance was observed in both the Ba-Bf and Mix groups, whereas the Bf group showed negligible changes over time. In the Ba-Bf group, the presence of *B. animalis* subsp. *lactis* provided a continuous supply of growth-supporting metabolites, enabling *B. fragilis* to maintain high and stable colonization levels throughout the 14-day period. In contrast, mice receiving the mixed metabolite solution exhibited a rapid but transient increase in *B. fragilis* abundance, followed by a progressive decline as metabolite availability diminished. Significant differences between groups emerged as early as day 2 and became more pronounced at later time points (Figure S9D). Moreover, analysis of overall microbial community structure using Bray–Curtis PCoA further supported these findings. The Ba-Bf group displayed gradual temporal shifts consistent with stable colonization and metabolic interaction between the two species. In the Mix group, however, day 7 and day 14 samples separated sharply from earlier time points, reflecting a pronounced but short-lived restructuring of the gut microbiota driven by the transient metabolite pulse (Figure S9E).

The results indicated that the high abundances of *Bacteroides* spp. were strongly associated with the colonization and secretion of specific metabolites of *B. animalis* subsp. *lactis* (Figure 4H), confirming the key role of *B. animalis* subsp. *lactis* as supportive bacteria in providing a suitable nutritional context for *Bacteroides*, the next nutrient-dependent bacterial colonizer.

### 2.6. Host inflammatory and mucosal responses to *B. animalis*–enhanced *B. fragilis* colonization

To assess whether the enhancement of *B. fragilis* colonization by *B. animalis* subsp. *lactis*—and the associated metabolites—affected host inflammatory status, we quantified serum IL-6, TNF-*α*, and IL-1β levels at the endpoint (day 14). As shown in Figure S8A–C, cytokine concentrations were highest in the control mice, whereas mice colonized with *B. animalis* subsp. *lactis* (Ba), *B. fragilis* (Bf), Ba-Bf (sequential *B. animalis* followed by *B. fragilis*), or the metabolite-Mix group (key metabolites followed by *B. fragilis*) displayed markedly lower levels of all three cytokines. Notably, the ability of *B. animalis* subsp. *lactis* to support and enhance *B. fragilis* colonization did not induce inflammation. These findings indicate that the cooperative colonization facilitated by *B. animalis* subsp. *lactis* is immunologically well tolerated and contributes to a less inflammatory gut environment.

Moreover, intestinal mucus layer staining with AB/PAS and quantification of the inner mucus layer in the large intestine after 14 d of colonization showed that neither *B. animalis* nor *B. fragilis* caused any reduction in inner mucus layer thickness (Figure S8D–E). In contrast, a pronounced increase in inner mucus layer thickness was observed in the Ba (15.7 ± 4 µm, *p* < 0.001) and Ba-Bf (10.9 ± 2 µm, *p* < 0.001) groups, whereas the Mix group (4.11 ± 1.2 µm, *p* = 0.5454) displayed values comparable to the control group. These findings suggest that *B. animalis* actively enhances intestinal mucus layer thickness and thereby supports *B. fragilis* colonization, while the metabolite mix promotes *B. fragilis* colonization without directly influencing host mucus layer integrity.

## 3. Discussion

This study demonstrates that *B. animalis* subsp. *lactis* and its derived metabolites can promote the colonization of *B. fragilis* in vitro but also in the gut of mice. Co-administration of the two species led to a significant increase in *B. fragilis* abundance, which was associated with key metabolites including 3-hydroxycapric acid, D-alanyl-D-alanine, 2-isopropylmalic acid, and D-glucose 2-phosphate. Hence, *B. animalis* subsp. *lactis* contributes to creating a metabolically favorable environment that supports *B. fragilis* establishment.

Gut microbial dynamics are shaped by a balance between competition and cooperation. While antagonistic interactions such as competition for limited resources can stabilize community diversity and prevent pathogen overgrowth, they may also hinder the establishment of beneficial microbes^9^ ^,^ ^10^ commensalism or mutualistic interactions, such as metabolite sharing, can play a critical role in sustaining microbial resilience.^11^ Early-colonizing genera like *Bifidobacterium*, *Collinsella*, *Ruminococcus*, and *Veillonella* are considered supportive bacteria that assist the establishment of later colonizers such as *Bacteroides* in the gut. Their absence, particularly in caesarean-delivered infants, is associated with reduced *Bacteroides* abundance.^12^ Our findings highlight a commensal interaction driven by metabolic cross‑feeding, where *B. animalis* subsp. *lactis* acts as a metabolically supportive species, providing key nutrients that enable robust *Bacteroides* colonization.^13^

Reflecting the ecological principle of cooperative interaction, microbial cross-feeding stands out as one of the major driving forces in shaping microbial communities, such as gut microbiota.^14^ *Bifidobacterium* species, for example, produce lactate that promotes the growth of butyrate-producing bacteria such as *Eubacterium hallii*, thereby contributing to gut health.^15^ ^,^ ^16^ Supplementation with *Bifidobacterium animalis* subsp. *lactis* BB-12 has been shown to modulate the gut microbiota, increasing the abundance of *Bacteroides*, *Lachnospiraceae*, and *Lactobacillus*).^17^ These shifts coincide with altered metabolic pathways related to amino acid and fatty acid biosynthesis, ultimately supporting overall gut function . Our findings demonstrate that *B. animalis* subsp. *lactis* promotes the growth of *Bacteroides* species, a process driven by its unique metabolite profile. Metabolites such as D-alanyl-D-alanine, N6-acetyl-L-lysine, and acetylglutamic acid act as essential sources of nitrogen, carbon, and sulfur, crucial for biomass formation and metabolic activity.^18^ Monosaccharides like D-glucose 2-phosphate and arabitol likely serve as direct carbon and energy sources, serving as substrates for glycolysis and the pentose phosphate pathway to generate pyruvate, a key intermediate in fermentation and energy production.^7^ Additionally, organic acids such as 2-isopropylmalic acid and 3-hydroxycapric acid may serve as alternative energy sources under carbohydrate-limited conditions.^19^ These metabolites are likely assimilated by *Bacteroides* through specialized transport systems, enabling more efficient nutrient acquisition and accelerated growth. Moreover, external acquisition of these compounds alleviates biosynthetic demands, conserving cellular resources and enhancing metabolic efficiency under nutrient-limited conditions.^20^ Hence, these metabolites serve as pivotal nutritional and energetic conduits between *B. animalis* subsp. *lactis* and *Bacteroides* spp., highlighting their cooperative metabolic interplay.

Early microbial colonizers play a pivotal role in shaping gut microbial community structure by orchestrating both the timing and availability of ecological resources. Our in vivo experiments demonstrate that *B. animalis* subsp. *lactis*, when introduced prior to *B. fragilis*, establishes itself effectively and enriches the gut environment with key metabolites, including 3-hydroxycapric acid, D-alanyl-D-alanine, 2-isopropylmalic acid, and D-glucose 2-phosphate. These compounds form a nutrient-rich environment that significantly enhances the growth of *B. fragilis*, with dose-dependent effects of metabolites and synergistic effects observed in both microbial co-colonization and metabolite-only supplementation groups. However, the timing of colonization proved critical: metabolite-only gavage induced a transient growth of *B. fragilis*, whereas early *B. animalis* presence supported sustained colonization of *B. fragilis*. This indicates not only the chemical but also the temporal aspects of microbial succession, where the sustained presence of the early-arriving taxa actively shapes the gut’s environment in ways that benefit nutrient-dependent successors such as *Bacteroides* spp.

Together, our in vitro and in vivo results demonstrate a commensal cross-feeding interaction between *B. animalis* subsp. *lactis* and *Bacteroides* spp., particularly under nutrient-limited conditions in which early *Bacteroides* colonization levels are significantly promoted by the presence of metabolically supportive taxa such as *Bifidobacteria* through secretion of functional substrates. This opens new avenues for translational applications where *B. animalis* subsp. *lactis* could be developed as a next-generation probiotic to support the establishment of beneficial taxa like *Bacteroides*, while its bioactive metabolites may serve as targeted prebiotics to promote a healthy and resilient gut microbiome during the critical early stages of life.

## 4. Methods and materials

### 4.1. Bacterial species and growth conditions

All bacterial species were acquired from the Chinese human Gut Microbial Biobank (hGMB - https://hgmb.nmdc.cn/). *Bifidobacterium animalis* subsp. *lactis* BNCC134318, *Bifidobacterium bifidum* BNCC186304, *Bifidobacterium breve* BNCC186529, *Bifidobacterium longum* BNCC185354*, Bacteroides dorei* DSM17855, *Bacteroides fragilis* BNCC314741*, Bacteroides ovatus* BNCC260545, *and Bacteroides thetaiotaomicron* BNCC354380 were routinely cultured in brain heart infusion (BHI, Merck, 53286) agar or broth (HopeBio, China, HB8297-1). Manipulation and incubation for all culture-based experiments were conducted in a sealed anaerobic chamber (Shanghai Longue Instrument Co., Ltd., China) at 37 °C with an atmosphere of 5% CO_2_, 5% H_2,_ and 90% N_2_. The medium was generally supplemented with 5% defibrinated sheep blood (Hopebio, China) or type II mucin (1%, Sigma-Aldrich, M2378) plus hemin (0.01 g/L, Merck, H9039) to facilitate the proper growth of *Bacteroides*. Growth was determined by OD_600_ using a plate reader (Biotek Synergy H1, Agilent, Santa Clara, USA).

### 4.2. In vitro cross-feeding challenges

Symbiosis and interactions of the *Bifidobacterium* spp. and *Bacteroides* spp. were assessed in three different levels of in vitro experiments as follows:

#### 4.2.1. Co-cross-streak plate assay

*Bifidobacterium* species (*n* = 4) and *Bacteroides* species (*n* = 4) were cultured individually overnight. The normalized bacterial solutions (OD_600_ = 0.35) were spread on BHI agar using aseptic cotton swabs. *Bifidobacterium* species were inoculated horizontally, while *Bacteroides* species were streaked vertically close to but not touching the edge of horizontal lines (Figure S1A). Plates were incubated anaerobically at 37 °C for 18–24 hours, and *Bacteroides* growth was compared to control plates streaked with *Bacteroides* only.

#### 4.2.2. Symbiosis on agar plate assay

To gain insight into the cell compartments that could promote the growth of the different *Bacteroides* species, the *Bifidobacterium* species were added individually to the reduced BHI broth (40 ml). Following overnight anaerobic incubation, 20 ml of the early exponential phase culture was directly frozen at −80 °C for 1 hour and thawed at 37 °C for 30 minutes (repeated 4 times), yielding the *Bifidobacterium* lysed solution (LYS). The remaining 20 ml was centrifuged at 10,000 × g for 20 minutes. Then, the supernatant was passed through the 0.22 μm syringe filter (Millipore SLGPR33RB) to prepare *Bifidobacterium* supernatant (SUP). At the same time, the pellet was resuspended in 10 ml of sterile PBS, the same freezing-thawing process (−80 °C for 1 hour and thaw at 37 °C for 30 minutes) was repeated 4 times, which represents the pellet group (PL). Notably, the successful lysis of *Bifidobacterium* by freeze/thaw was checked by spotting the solution on BHI + 5% sheep blood or type II mucin (1%) plus hemin (0.01 g/L). The modified minimal medium (MM)^21^ [for 1000 ml: (NH_4_)_2_SO_4_, 1 g; KH_2_PO_4_, 0.9 g; NaCl, 0.9 g; CaCl_2_ · 2 H_2_0, 26.5 mg; MgCl_2_ · 6 H_2_0, 20 mg; MnCl_2_ · 4 H_2_0, 10 mg; FeSO_4_ · 7 H_2_0, 4 mg; CoCl_2_ · 6 H_2_0, 1 mg; hemin, 4 mg and 20 g agar for agar plates] was supplemented with the LYS, PL, or SUP suspensions at a 1:1 (v/v) ratio. A mixture of MM and BHI broth was used as control. Then, the agar plates were inoculated with *Bacteroides* spp., kept in anaerobic conditions at 37 °C for 24-36 hours, and the growth of *Bacteroides* spp. were compared to control plates (MM plates streaked with *Bacteroides* spp. only).

#### 4.2.3. Symbiosis in the broth assay

To assess the symbiosis between *Bifidobacterium* spp. and *Bacteroides* spp., MM was supplemented with *Bifidobacterium animalis* subsp. *lactis* solutions of LYS, PL, SUP, or BHI (control), and after 24 hours of anaerobic pre-incubation, the media were individually inoculated with early-exponential cultures (OD_600_ = 0.6) of *B. dorei, B. fragilis, B. ovatus,* and *B. thetaiotaomicron*. Samples were collected at multiple time points (up to 24 h). *Bacteroides* spp. growth was assessed primarily by measuring sample turbidity (OD₆₀₀), using SUP or LYS solutions without *Bacteroides* inoculation as the blank for background correction. The genomic DNA yielded from 1 ml samples was used as a proxy to measure biomass and reported as ng/µl. Moreover, *Bacteroides* growth was quantified using qPCR on genomic DNA extracted from 1 ml of samples, as explained in Section 4.3.

#### 4.2.4. Physicochemical and biochemical treatments of *B. animalis* supernatant

*B. animalis* supernatant (prepared as described in Section 4.2.2) was subjected to the following treatments individually to assess the relative contribution of pH, redox state, heat-labile components, peptide/proteinaceous factors, hydrophobic metabolites, and molecular weight fractions to its overall biological activity. To exclude pH dependent effects, the pH of the supernatant was adjusted to match the control medium (pH ≈ 7.0) using sterile 1 M HCl or NaOH. In parallel, a buffered supernatant was prepared using 50 mM HEPES (Sigma-Aldrich, H0887) to maintain stable pH during incubation. To eliminate nonspecific redox effects, the oxidation–reduction potential (ORP) of the supernatant was adjusted to the physiological anaerobic range (–350 to –400 mV) using sterile reducing agents (e.g., cysteine-HCl) under anaerobic conditions. ORP was monitored using a microredox electrode (METTLER, InPro 3100-i), and supernatants were equilibrated for 30 min before use. To inactivate heat-labile components, aliquots of the supernatant were incubated at 95 °C for 10 min or at 65 °C for 60 min. These treatments denature proteins, peptides, and enzymelike factors. To remove peptide-based or proteinaceous molecules, supernatant aliquots were treated with Proteinase K (100 µg/mL) at 55 °C for 30 min. Reactions were terminated by heating at 95 °C for 10 min. This treatment degrades quorum sensing peptides, small bioactive peptides, and larger proteinaceous factors. To remove hydrophobic and aromatic metabolites, sterile activated charcoal (10 mg/mL) was added to the supernatant and incubated with gentle agitation for 30 min at room temperature. Samples were centrifuged at 12,000 × g for 5 min and passed through a 0.22 µm syringe filter. This treatment removes bile acids, aromatic metabolites, hydrophobic lipids, and other nonpolar compounds. For molecular weight fractionation, the supernatant was processed using Amicon Ultra centrifugal filters with a 3 kDa molecular weight cutoff. Two fractions were collected: the <3 kDa fraction, enriched for small molecule metabolites (organic acids, amino acids, sugars, cofactors), and the >3 kDa fraction, enriched for peptides, proteins, polysaccharides, and other macromolecules. Both fractions were sterilized by 0.22 µm filtration before use. All treated and untreated supernatants were used immediately for parallel growth assays.

#### 4.2.5. Growth curve modeling in the presence of metabolites

To explore the supportive potential of selected metabolites produced exclusively by *B. animalis* and significantly consumed by *B. fragilis*, mouse cecal fluid from untreated animals was used as the basal medium to minimize confounding effects from exogenous compounds and to closely mimic the native metabolic environment of the gut, thereby providing a physiologically relevant context for assessing metabolite‑dependent effects. The mice cecum fluid was supplemented using 5 mM 3-hydroxycapric acid (Sigma-Aldrich, H3648), 6 mM D-**a**lanyl-D-alanine (Sigma-Aldrich, A0912), 4 mM D-glucose 2-phosphate (Synthose Inc, DG615) and 1 mM 2-isopropylmalic acid (Sigma-Aldrich, 333115) individually or as a mixture of all metabolites at the mentioned concentration. Briefly, cecal contents from untreated mice (ctrl group) were resuspended in reduced PBS at a ratio of 0.1 g per 1 mL. The suspension was homogenized thoroughly, followed by centrifugation at 10,000 × g to remove particulate matter. The resulting supernatant was then passed through a 0.22-µm syringe filter to obtain sterile cecum fluid. The prepared media were dispersed to Sterilin™ Clear Microtiter™ Plates (612F96). Growth of *B. fragilis* under anaerobic conditions and 37 °C was monitored by recording the OD_600_ in a Stratus microplate reader (Cerillo, Charlottesville, VA, USA) shaking on a Microplate Shaker PMS-1000i (Grant Instruments, Shepreth, Cambridgeshire, UK) at 180 rpm. All experiments were conducted using the default kinetic mode. Cecal fluid from untreated mice served as the negative control, and its OD measurements were used to subtract background noise from the experimental readings.

### 4.3. qPCR to assess *Bifidobacterium* and *Bacteroides* growth

The abundance of bacteria was assessed using genus-specific primers bac-F: GGARCATGTGGTTTAATTCGATGAT and bac-R: AGCTGACGACAACCATGCAG (amplicon size ≅ 124 bp) for *Bacteroides* spp., and bif1-F: AGGGTTCGATTCTGCTCAG and bif1-R: CATCCGGCATTACCACCC (amplicon size ≅ 510 bp) for *Bifidobacterium* spp.^22^ using PC33-2X SYBR Green qPCR Mix (AID LAB) following the manufacturer’s protocols. Samples (1 ml) were processed with the genomic DNA extraction Kit (E.Z.N.A. Omega Bio. Tek) according to the manufacturer’s protocol with minor modifications, including two steps of cell lysis using lysozyme (50 ng) and vortexed 20 min with 0.2 mm glass beads. DNA concentration was determined by Qubit^TM^ 4 Fluorometer (Thermo Fisher Scientific, Massachusetts, USA) and qPCR was performed on a dilution of 1:10 using the Thermo Fisher ABI QuantStudio 1 Real-Time PCR machine. CT values were converted to copy numbers using a standard curve generated from qPCR amplification of purified *B. animalis* subsp. *lactis* or *B. dorei.* Results were visualized in copies/μL. The limit of detection (LOD) and limit of quantification (LOQ) for *B. animalis* qPCR were estimated at ~9 and ~10^3^ copies, respectively, and for *B. fragilis* at ~50 and ~10^3^ copies, based on consistent Ct detection and linearity across replicates.

### 4.4. Metabolomics analysis

To assess metabolite profiles, supernatants from 36-hour anaerobic cultures of *Bifidobacterium* spp. and *Bacteroides* spp. grown on BHI broth were collected. Additionally, *Bacteroides* spp. were incubated in filtered supernatants derived from *B. animalis* cultures for 24 hours under anaerobic conditions at 37 °C. Six biological replicates per condition, along with fresh BHI broth as control, were snap-frozen at −80 °C prior to extraction. For metabolite isolation, 100 µL of each supernatant was mixed with 400 µL of chilled methanol:acetonitrile (1:1, v/v), vortexed, and sonicated for 1 hour in an ice bath. Samples were then incubated at −20 °C for 1 hour, followed by centrifugation at 14,000 × g for 20 minutes at 4 °C. The resulting supernatants were dried under vacuum and stored for LC-MS analysis.

#### 4.4.1. UHPLC-MS/MS analysis

Metabolomic profiling was performed using a Shimadzu Nexera X2 UHPLC system coupled to a Q-Exactive Plus Orbitrap mass spectrometer (Thermo Scientific). Chromatographic separation utilized an ACQUITY UPLC® HSS T3 column (2.1 × 100 mm, 1.8 μm; Waters) with a flow rate of 0.3 mL/min. The mobile phases consisted of 0.1% formic acid in water (A) and acetonitrile (B), with a gradient elution from 0% to 100% B over 10 minutes, followed by re-equilibration. Mass spectrometry was conducted in both positive and negative electrospray ionization modes. Instrument parameters included spray voltages of 3.8 kV (positive) and 3.2 kV (negative), capillary temperature of 320 °C, and probe heater temperature of 350 °C. Full MS scans were acquired over m/z 70–1050 at 70,000 resolutions, with MS/MS scans at 17,500 resolution using stepped collision energies of 20, 30, and 40.

#### 4.4.2. Data preprocessing and filtering

Raw spectral data were processed using MS-DIAL software for peak detection, alignment, and normalization. Metabolite annotation was based on accurate mass (tolerance < 10 ppm) and MS/MS fragmentation patterns (tolerance < 0.02 Da), cross-referenced against HMDB, MassBank, and an in-house spectral library. Features with nonzero values in at least 50% of samples within any group were retained for downstream analysis.

#### 4.4.3. Comparative analysis

Fold changes were calculated for the paired comparisons. For each pair, the fold change of a given metabolite was determined by dividing the mean metabolite abundance of the first group by the mean abundance of the second group in the specified comparison. For in vitro experiments, fold changes were calculated using the mean values of six biological replicates per group, whereas for in vivo experiments, calculations were based on five biological replicates per group. Raw metabolite measurements for all replicates are provided in Table S2. As fold changes were derived from group means rather than individual measurements, the bar charts are presented in arbitrary units (fold change) and are shown without standard deviation or error bars. Statistical significance was assessed using the original replicate values for each paired comparison, and the resulting *P*-values are reported as significance levels.

#### 4.4.4. Pathway enrichment

Differentially abundant metabolites were mapped to biological pathways using the KEGG database. Enrichment analysis was performed using Fisher’s exact test, with false discovery rate (FDR) correction applied to account for multiple comparisons. Pathways with adjusted *p*-values below 0.05 were considered significantly enriched.

### 4.5. In vivo experiment

Animal experiments were approved by the Ethical Committee of Guangdong Provincial People’s Hospital (Ethical Approval No. KY2023-878-01). The 8–12-week-old male and female C56BL/6 mice were kept in cage setups with autoclaved food and water to minimize cross-contamination. All mice were primarily administered an antibiotic cocktail (AVNM) of ampicillin (1 g/L), vancomycin (0.5 g/L), neomycin (1 g/L), and metronidazole (1 g/L) (all from Sigma-Aldrich) for two weeks.^23^ Elimination of the microbiota was tracked by anaerobic plating on BHI plates supplemented with 5% sheep blood. Antibiotic treatment was stopped 12 hours before subjecting the mice to any test. The mice were randomly assigned to 5 groups (five mice per cage). *B. fragilis* was cultured anaerobically to early exponential phase, washed, and resuspended in pre‑reduced PBS. Based on our standard calibration of OD_600_ versus CFU for this strain and the dilution scheme used, the gavage suspension contained approximately 3 × 10^8^ CFU/mL. *Bifidobacterium* culture was also prepared for the same final CFU/mL. Mice were orally administered 200 µl of bacterial solutions (∼6 × 10^7^ CFU per mouse), including *B. animalis* subsp. l*actis* at time 0 (Ba group), *B. fragilis* at time 12 (Bf group), *B. animalis* subsp. *lactis* at time 0 and then *B. fragilis* at time 12 (Ba-Bf group). Mice assigned to the Mix group received a daily oral gavage of 200 µL of a metabolite solution containing 5 mM 3-hydroxycapric acid (Sigma-Aldrich, H3648), 6 mM D-alanyl-D-alanine (Sigma-Aldrich, A0912), 4 mM D-glucose 2-phosphate (Synthose Inc., DG615), and 1 mM 2-isopropylmalic acid (Sigma-Aldrich, 333115). To contextualize the in vivo dosing strategy, we conducted a comparative analysis of structurally related metabolites reported in the literature. The selected doses (10 and 40 mg/kg) fall within the range of previously published studies employing oral gavage^24–27^ and were chosen to ensure sufficient bioavailability and biological activity given the uncertainty of intestinal absorption and host metabolism. In parallel, the in vitro concentrations were determined based on the measured relative abundance of these metabolites in culture supernatants and fecal samples (Table S2), and their physiological relevance was further supported by dose–response experiments demonstrating growth enhancement in the presence of represented concentration of the metabolites (Figure 4E and Section 4.2.5). Time-course analyses (Figure 4F) additionally illustrate the dynamic microbial response to metabolite exposure. The 14-day treatment period was chosen to allow sufficient time for microbial and metabolic adaptation while minimizing potential confounding effects of long-term exposure. Fresh fecal samples were collected into sterilized tubes at designated time points throughout the 14-day experimental period. Mice were monitored daily for general health status, behavior, and body weight to ensure animal welfare and to detect any treatment-related adverse effects. Collected fecal pellets were resuspended in 500 µL PBS, homogenized, and processed either for qPCR analysis to quantify the copy number of *B. fragilis* and *B. animalis* subsp. *lactis* per gram of feces, or for metabolomic profiling to measure the levels of metabolites.

### 4.6. Mucin layer feature analysis

Mucin layer morphology was assessed to evaluate host level responses associated with *B. fragilis* colonization in the presence or absence of *B. animalis*. At the experimental endpoint, distal colon tissues were collected from mice in all tested groups, ensuring that mucin layer features were examined across the full set of colonization conditions. Tissues were fixed in 4% paraformaldehyde at 4 °C overnight, dehydrated through an ethanol gradient, embedded in paraffin, and sectioned at 5 µm thickness using a rotary microtome. For visualization of the mucin layer, sections were stained using alcian blue–periodic acid schiff (AB-PAS) following standard protocols. Briefly, slides were deparaffinized, rehydrated, and incubated with 1% alcian blue (pH 2.5) to label acidic mucins, followed by periodic acid oxidation and Schiff reagent to detect neutral mucins. Slides were counterstained with hematoxylin, dehydrated, and mounted with resinous medium. Images were acquired using a brightfield microscope (Evident Olympus VS200) equipped with a digital camera. For each mouse, at least five nonoverlapping fields were imaged at 20 × or 40 × magnification. Mucin layer thickness and structural integrity were quantified using ImageJ (NIH) by measuring the distance from the epithelial surface to the outer mucus boundary. All analyses were performed in a blinded manner.

### 4.7. Quantification of inflammatory cytokines

Serum concentrations of inflammatory cytokines were measured at day 0 (baseline) and day 14 (endpoint). Blood samples were allowed to clot at room temperature for 30 min and centrifuged at 3,000 × g for 10 min to isolate serum. All samples were stored at −20 °C until analysis. Levels of IL-1β (Proteintech, Cat. No. KE10003), TNF-*α* (Proteintech, Cat. No. KE10002), and IL-6 (Proteintech, Cat. No. KE10007) were quantified using commercially available mouse ELISA kits. All assays were performed according to the manufacturer’s instructions. Briefly, standards and appropriately diluted serum samples were added to pre-coated 96-well plates and incubated for the recommended duration. After washing, wells were incubated with biotin-conjugated detection antibodies followed by HRP-streptavidin. Color development was achieved using TMB substrate, and reactions were stopped with sulfuric acid. Absorbance was measured at 450 nm using a microplate reader (BIORAD-PR 4100). Cytokine concentrations were calculated from standard curves generated for each assay. Data from day 0 and day 14 were compared to evaluate treatment-associated changes in inflammatory status.

### 4.8. Strain background and genotypic confirmation of non-toxigenic *Bacteroides* spp

Whole-genome sequencing was performed using both Illumina and Nanopore platforms, followed by *de novo* hybrid assembly to obtain a high-quality reference genome of all bacterial species used in the study. To confirm the non-toxigenic status of the strain, a comprehensive genotypic screen was conducted to assess the presence or absence of *Bacteroides* toxin (BFT) genes. The assembled genome was queried using tblastn (v2.16.0+) against a curated database of all known BFT protein sequences obtained from UniProtKB, including bft-1, bft-2, bft-3, and related variants. Searches were performed using an E-value threshold of 1 × 10.^1^ No significant hits were detected for any BFT-encoding genes, confirming that *B. fragilis* BNCC314741 is non-toxigenic (ETBF-negative). This genotypic verification ensures strain safety and supports reproducibility and interpretability of all downstream experiments.

### 4.9. 16S rRNA metataxonomic analysis

Total genomic DNA was extracted from mouse fecal samples using the *QIAamp DNA Stool Mini Kit* (Qiagen), following the manufacturer’s protocol. The V3–V4 hypervariable regions of the bacterial 16S rRNA gene were amplified using universal primers 341F (5′-CCTACGGGNGGCWGCAG-3′) and 806R (5′-GACTACHVGGGTATCTAATCC-3′). Amplicons were purified, quantified, and pooled in equimolar concentrations prior to sequencing on the Illumina *MiSeq* platform. Raw reads were processed using the DADA2 pipeline, including quality filtering, denoising, paired-end merging, and chimera removal to generate high-resolution amplicon sequence variants (ASVs). Taxonomic classification was performed using a Naive Bayes classifier trained on the SILVA v138.1 (nr99) database. To account for differences in sequencing depth, the ASV table was rarefied to a uniform sampling depth. To evaluate the impact of *B. animalis* and *B. fragilis* colonization on the gut microbiota over time, samples were group Bf, Ba_Bf, and Mix were analyzed across five time points (day 0, 1, 2, 7, and 14). Principal Coordinates Analysis (PCoA) based on Bray–Curtis dissimilarity was used to visualize temporal shifts in microbial community composition within each group. Additionally, longitudinal changes in the relative abundance of key taxa, including *Bacteroides fragilis*, were quantified and statistically compared across conditions and time points using appropriate non-parametric tests.

### 4.10. Statistical analysis

Multivariate analyses were conducted in R (v4.0.3), employing Pareto scaling and dimensionality reduction via PCA, PLS-DA, and OPLS-DA. Growth kinetics and gene copy number data were analyzed in GraphPad Prism (v9.1.1). Group comparisons were evaluated using two-tailed Student’s t-tests or Kruskal–Wallis tests, as appropriate. ANOVA and two-way ANOVA were applied for multi-group and interaction analyses. Detailed statistical outputs, including exact *p*-values and test statistics, are provided in the supplementary tables.

## Supplementary Material

Supplementary figures.pdf Supplementary figures.pdf

Table S3.xlsx Table S3.xlsx

Table S2.xlsx Table S2.xlsx

Table S1.xls Table S1.xls

## Data Availability

Genomic datasets, including all raw sequence data for *Bifidobacterium* and *Bacteroides* spp., have been deposited in the GenBank database under BioProject PRJNA1043735. The 16S metataxonomic raw sequencing data are available under accession numbers SAMN54923384–SAMN54923457 (BioProject PRJNA1415141). Metabolomics data generated from *Bifidobacterium* spp. supernatants are provided within the main article and in the Supplementary file (Table S2).

## Abbreviations

AB-PAS: Alcian Blue–Periodic Acid Schiff
ANOVA: Analysis of Variance
AVNM: Ampicillin, Vancomycin, Neomycin, Metronidazole (antibiotic cocktail)
BHI: Brain Heart Infusion
CFU: Colony Forming Unit
ctrl: Control
DNA: Deoxyribonucleic Acid
EPS: Exopolysaccharides
ETBF: Enterotoxigenic *Bacteroides fragilis*
FDR: False Discovery Rate
Fig: Figure
hGMB: human Gut Microbial Biobank
HRP: Horseradish Peroxidase
IL-1β, IL-6: Interleukin 1 beta, Interleukin 6
IFNγ: Interferon gamma
LCFA: Long-Chain Fatty Acids
MCFA: Medium-Chain Fatty Acids
MM: Minimal Medium
MS/MS: Tandem Mass Spectrometry
MSI: Metabolomics Standards Initiative
OD_600_: Optical Density at 600 nm
ORP: Oxidation–Reduction Potential
PBS: Phosphate Buffered Saline
PC1, PC2: Principal Component 1 and 2
PCA: Principal Component Analysis
PCR: Polymerase Chain Reaction
qPCR: Quantitative Polymerase Chain Reaction
PLS-DA: Partial Least Squares Discriminant Analysis
OPLS-DA: Orthogonal PLS-DA
SCFA: Short-Chain Fatty Acids
SEM: Standard Error of the Mean
TCA: Tricarboxylic Acid (cycle)
TMB: 3,3′,5,5′-Tetramethylbenzidine
TNF-α: Tumor Necrosis Factor alpha
UHPLC-MS/MS: Ultra-High Performance Liquid Chromatography–Tandem Mass Spectrometry

## Abbreviation used in this manuscript only

Ba or ba: *Bifidobacterium* *animalis*
bb: *Bifidobacterium bifidum*
Bf or fra: *Bacteroides fragilis*
BFT: *Bacteroides toxin*
bl: *Bifidobacterium* *longum*
bbr: *Bifidobacterium* *breve*
dor: *Bacteroides dorei*
ova: *Bacteroides ovatus*
thet: *Bacteroides thetaiotaomicron*
LYS: *Bifidobacterium* *animalis* lysed broth culture
SUP: The supernatant part of *Bifidobacterium* *animalis* culture after centrifugation
PL: Only the lysed pellets of *Bifidobacterium* *animalis* after centrifugation and resuspension in sterile PBS
