# Supplementary material for: A metabolite-dependent mechanism by which Bifidobacterium animalis subsp. lactis promotes Bacteroides colonization
Source: Gut Microbes. 2026 Jul 7;18(1):2696647. doi: 10.1080/19490976.2026.2696647 (PMC13348967; doi:10.1080/19490976.2026.2696647)
Supplement: Supplementary figures.pdf [file KGMI_A_2696647_SM3876.pdf]

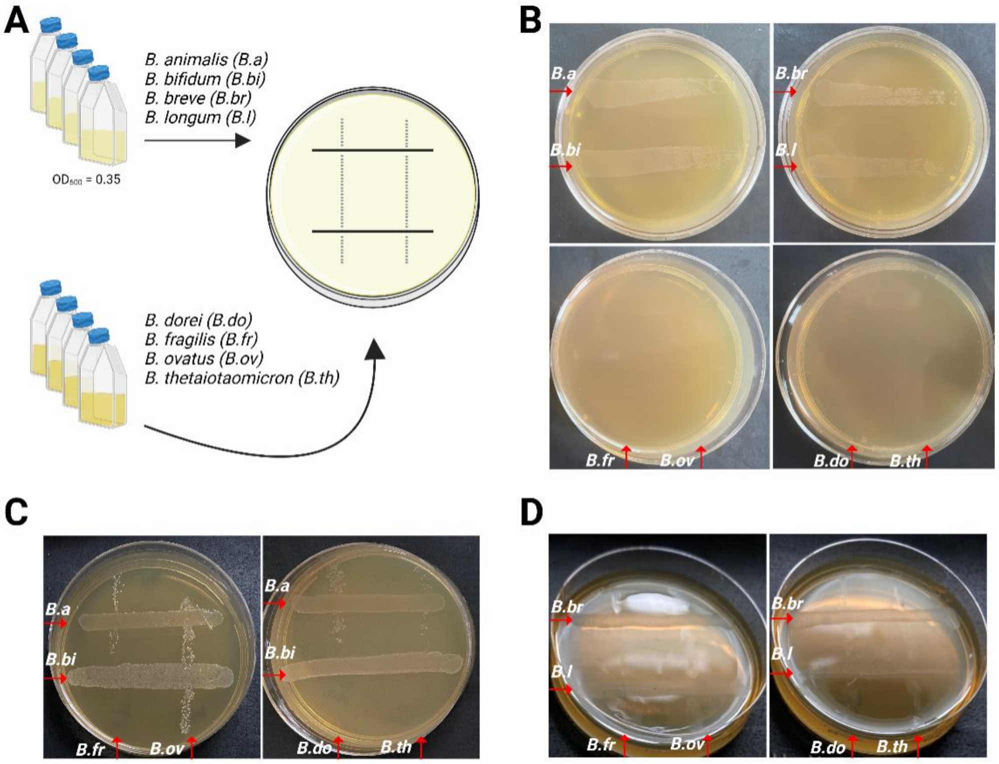

Fig S1. Synergistic growth of *Bifidobacterium* spp. and *Bacteroides* spp. on BHI agar plates. (A) Experimental setup. (B) *Bifidobacterium* spp. grew on the agar plates in strictly anaerobic conditions, while *Bacteroides* spp. alone did not grow even after 72 h incubation. (C) *B. animalis* supported the colony formation of *B. dorei, B. fragilis, B. ovatus,* and *B. thetaiotaomicron,* while *B. bifidum* only partially supported the growth of *B. ovatus*. (D) No *Bacteroides* spp. colonies were detected on co-cultivation plates testing for synergy with *B. breve* and *B. longum*. Acronyms used: B.a, *B. animalis;* B.b, *B. bifidum;* B.br, *B. breve; B.l, B. longum;* B.do, *B. dorei;* B.fr, *B. fragilis;* B.ov, *B. ovatus;* B.th, *B. thetaiotaomicron.*

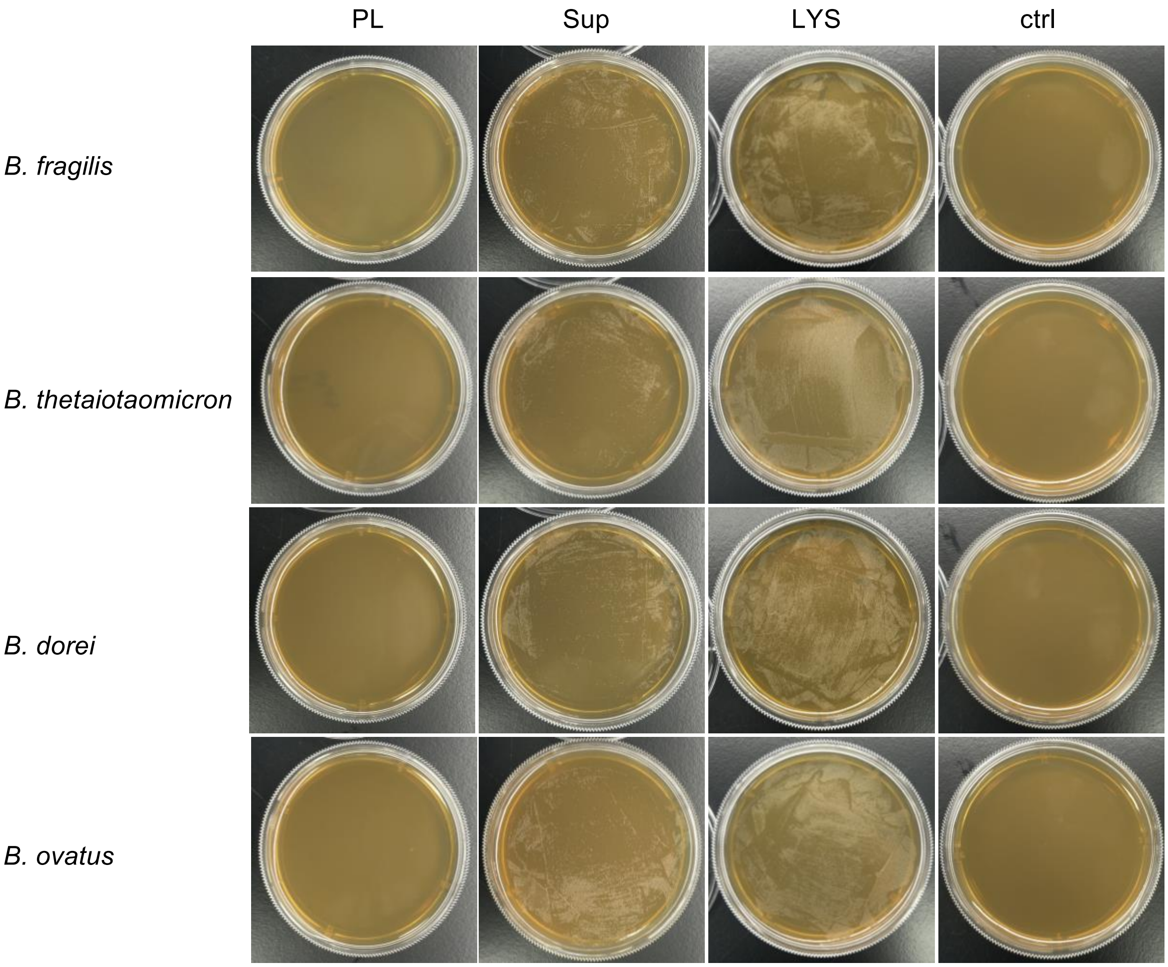

Fig S2. Related to **Fig 1**. (B) Growth of *B. dorei, B. fragilis, B. ovatus,* and *B. thetaiotaomicron* on agar plates supplemented with *B. animalis*-derived supernatant (SUP) and lysed pellet (LYS). Colony growth was evident only in the presence of SUP and LYS.

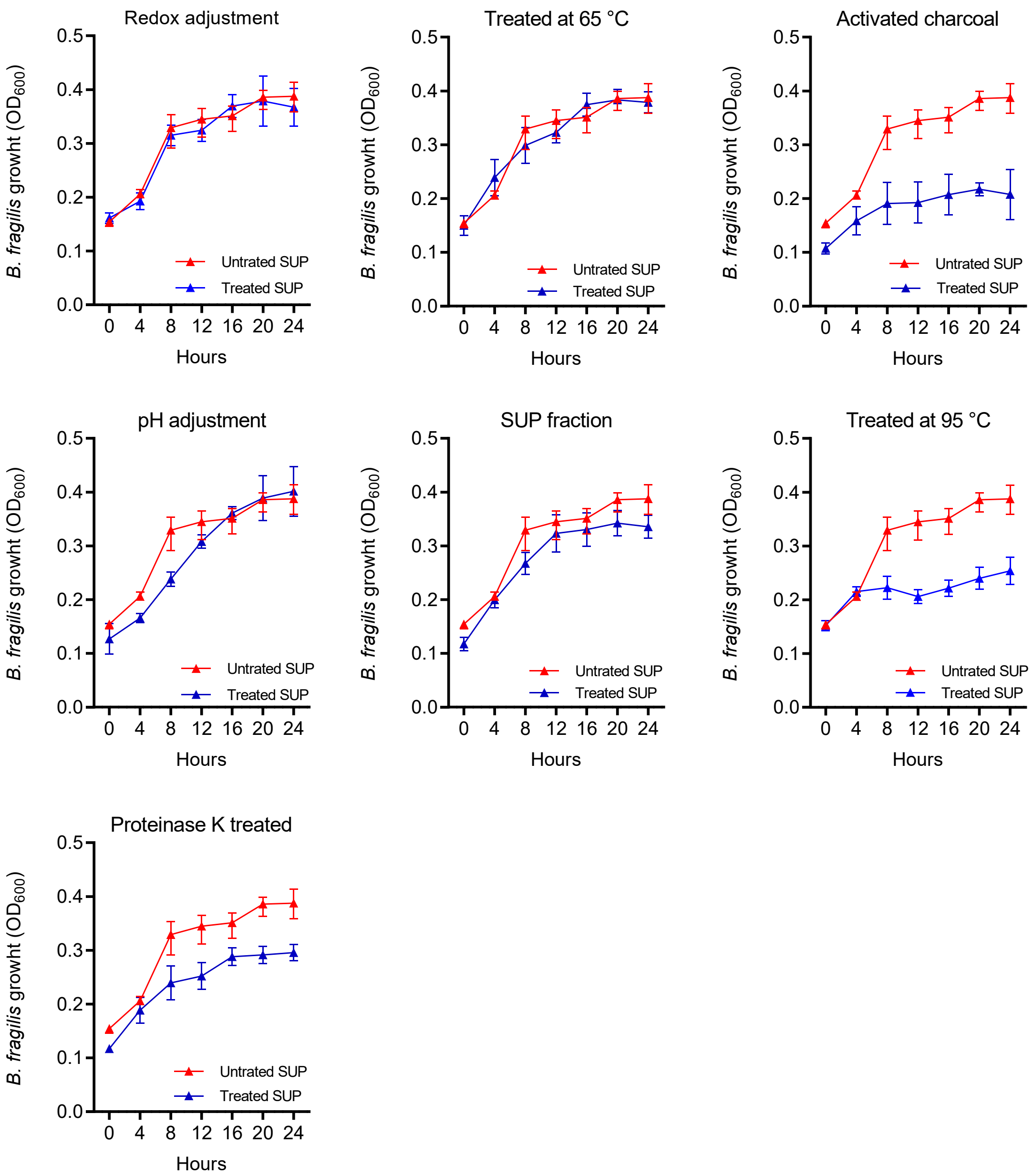

Fig S3. Growth of *Bacteroides fragilis* in response to physicochemically and biochemically treated *B. animalis* subsp. *lactis* supernatant (SUP). OD_{600} measurements over 24 h show *B. fragilis* growth in the presence of untreated SUP (red) versus treated SUP (blue) under anaerobic conditions. Treatments included redox adjustment, heat exposure (65 °C and 95 °C), activated charcoal extraction, pH neutralization, molecular weight fractionation (<3 kDa vs >3 kDa), and Proteinase K digestion. Each treatment selectively disrupted specific molecular features of the supernatant. Error bars represent standard deviation from biological replicates (n = 3).

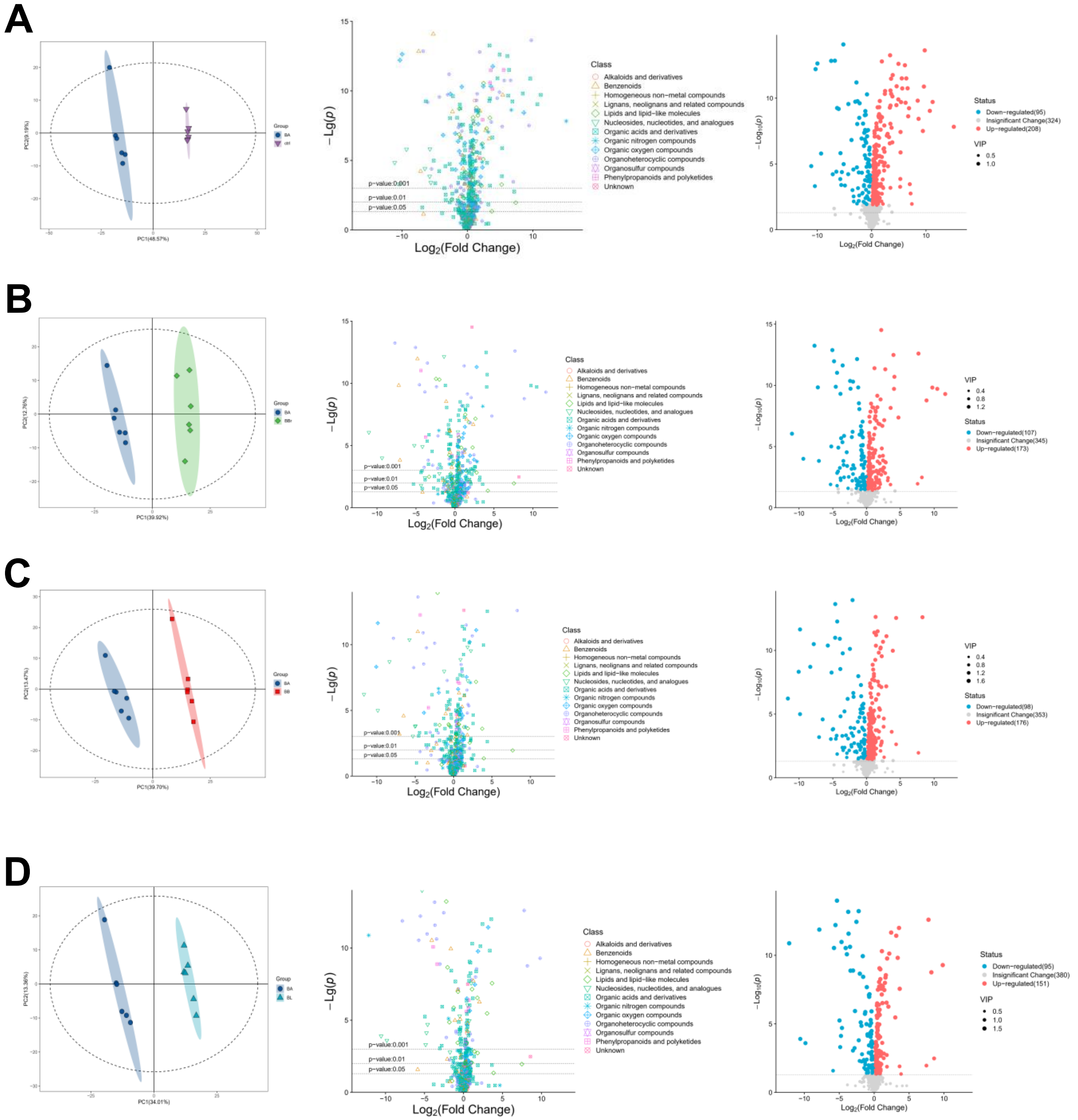

Fig S4. Related to **Fig 2**. Differentially abundant production and depletion of diverse metabolites by *B. animalis* compared with (A) control, (B) *B. breve*, (C) *B. bifidum,* and (D) *B. longum*. PCA plot of the metabolomics profile based on PC1 and PC2 from mean intensity values of total detected metabolites (Log_2 Fold Change). Volcano plots of differentially abundant metabolite features compared with each group. P-values are based on Benjamini–Hochberg corrected Welch's t tests. Features are colored based on their function. Significant production or depletion was defined based on changes in the scale of linear regression models of peak area by tested groups (changes in metabolite abundance), with FDR-adjusted p-value < 0.1 and log2FC > 0.5.

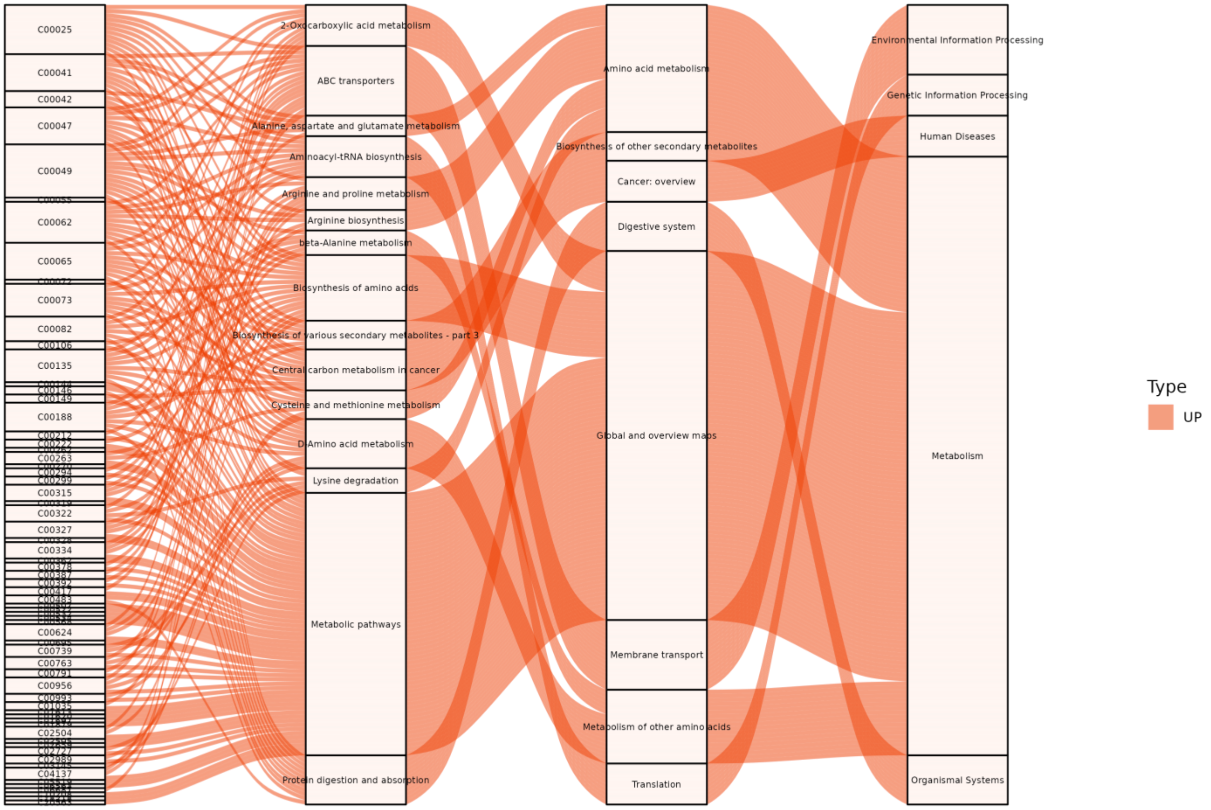

Fig S5. Related to **Fig 2**. KEGG pathway of *B. animalis*. The figure shows the identified metabolic pathways and enzymes involved in the utilization and production of various carbohydrates, amino acids, and organic acids. In *B. animalis*. The figure also shows the biosynthesis of some important metabolites, such as short-chain fatty acids, lactate, and exopolysaccharides, by *B. animalis*. The figure is based on the KEGG database and the genome annotation of *B. animalis.*

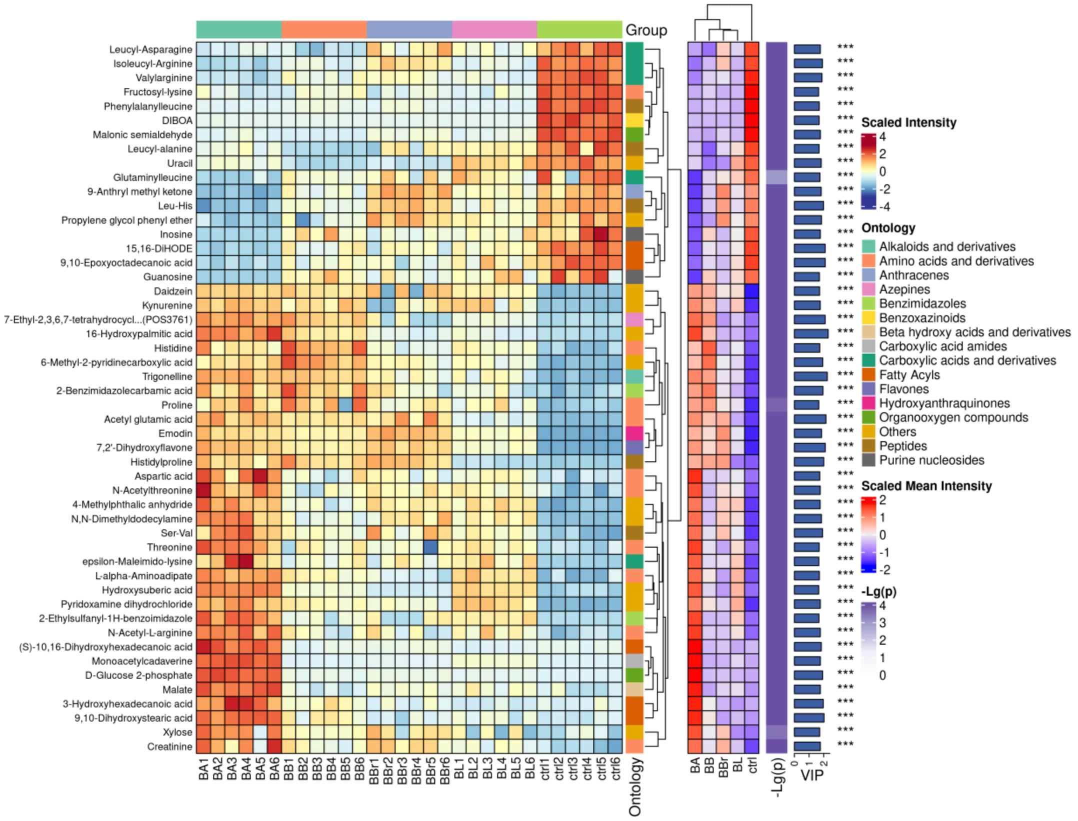

Fig S6. Related to **Fig 4**. Complex heatmap showing the annotated chemical abundances in six biological replicates of metabolomics for *B. animalis, B. breve, B. bifidum, B. longum* and control. Annotated chemical abundances in six biological repetitions of metabolomics for *B. animalis, B. breve, B. bifidum, B. longum* and control (**Table S4**). The figure shows a heatmap of the relative abundances of the metabolites that were detected and annotated in the supernatant of *Bifidobacterium* culture or the control group (media alone). The metabolites are grouped into 16 categories (ontology) based on their chemical classes. The samples are clustered based on their metabolic profiles, using hierarchical clustering and Euclidean distance. The color scale indicates the log-transformed and normalized abundances of the metabolites, ranging from blue (low) to red (high). The figure reveals the distinct metabolic signatures of each *Bifidobacterium* spp., as well as the common and unique metabolites that they produce or consume. The figure also shows the correlation between the metabolites and the *Bifidobacterium* spp., using Pearson's correlation coefficient and p-value (-log p).

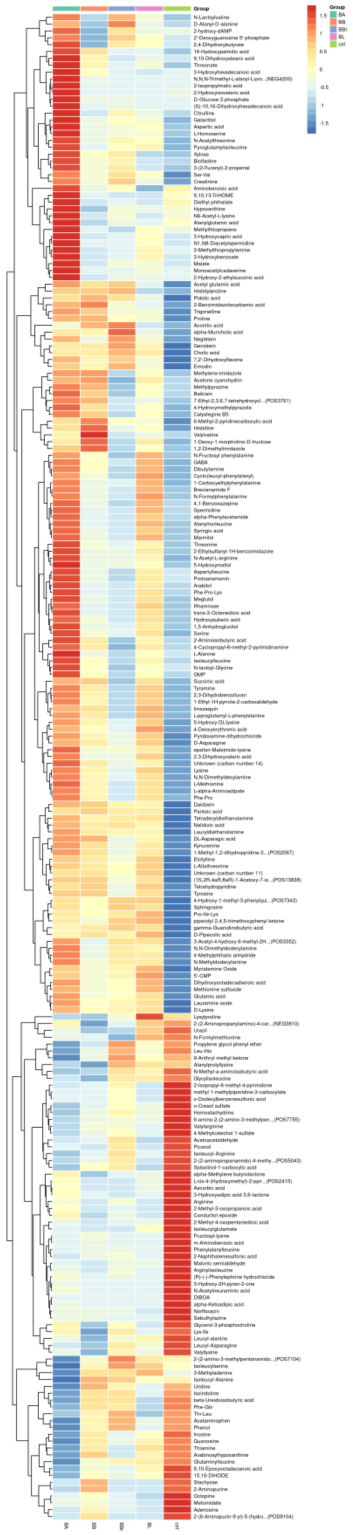

Fig S7. Related to **Fig 2**. Heatmap of individual key metabolites in cultures *B. animalis* subsp. *lactis, B. bifidum, B. breve*, *B. longum,* and the control. Features shown are those whose abundance significantly differed from controls (FDR-adjusted p < 0.1 and absolute log2 fold change >0.75). BA, *Bifidobacterium animalis.* BB, *Bifidobacterium bifidum.* BL*, Bifidobacterium longum. BBr, Bifidobacterium breve.*

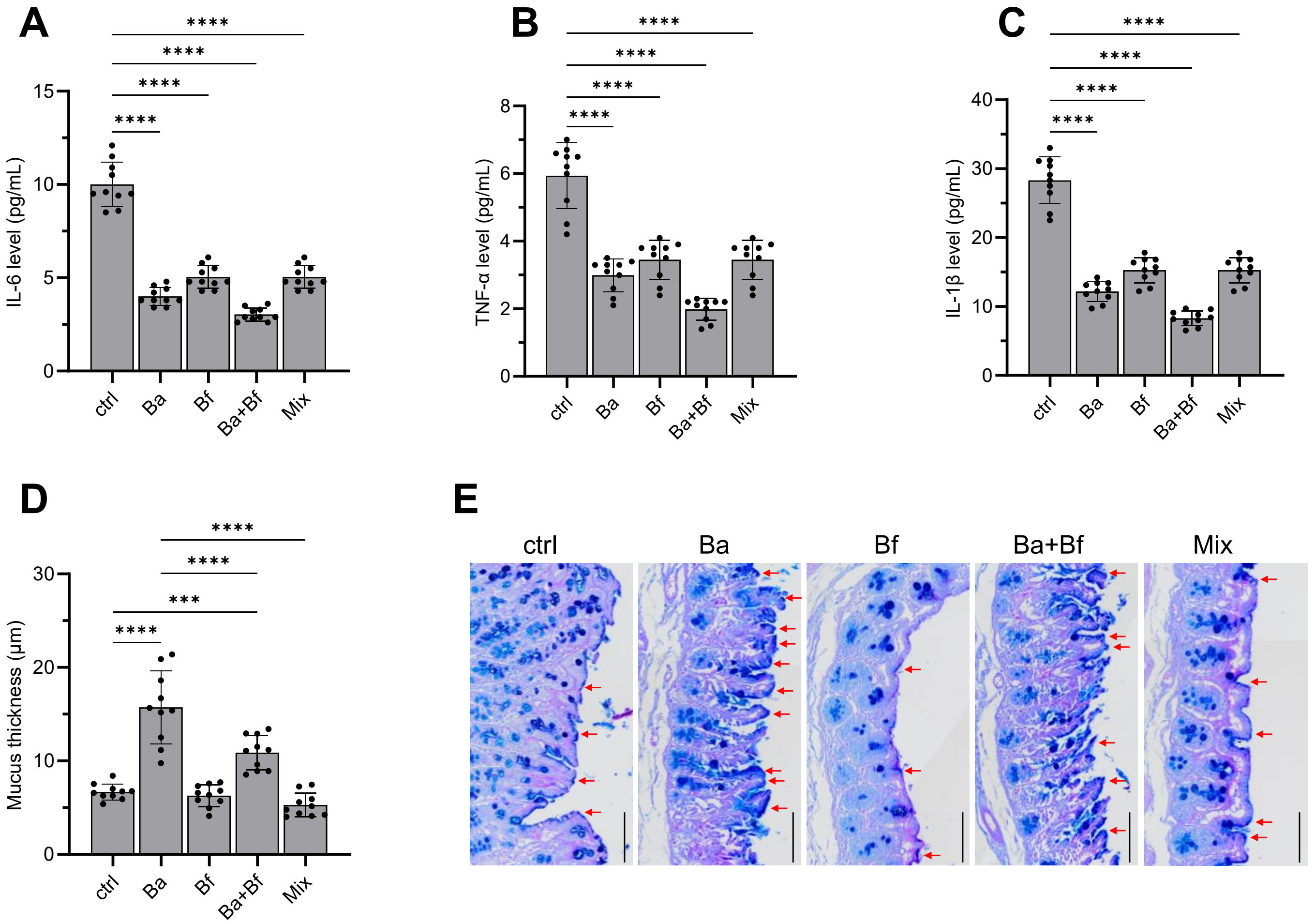

Fig S8. Cytokine levels (pg/mL) and colon mucus barrier on day 14 across experimental groups. A, IL-6, B, TNF-a, and C, IL-1ß concentrations were significantly elevated in the control group compared to all treatment groups. D, effect of tested bacterial colonization on the thickness of the mucus layer of mice. E. AB-PAS staining of proximal colon tissue (magnification: 40×5). Red arrows indicate the colonic mucus layer. Scale bar = 100 μm. Statistical significance is indicated by asterisks (***$p < 0.001$, ****$p < 0.0001$). Data are presented as mean ± SEM with individual values shown; n = 5 mice per group, with each sample measured in duplicate.

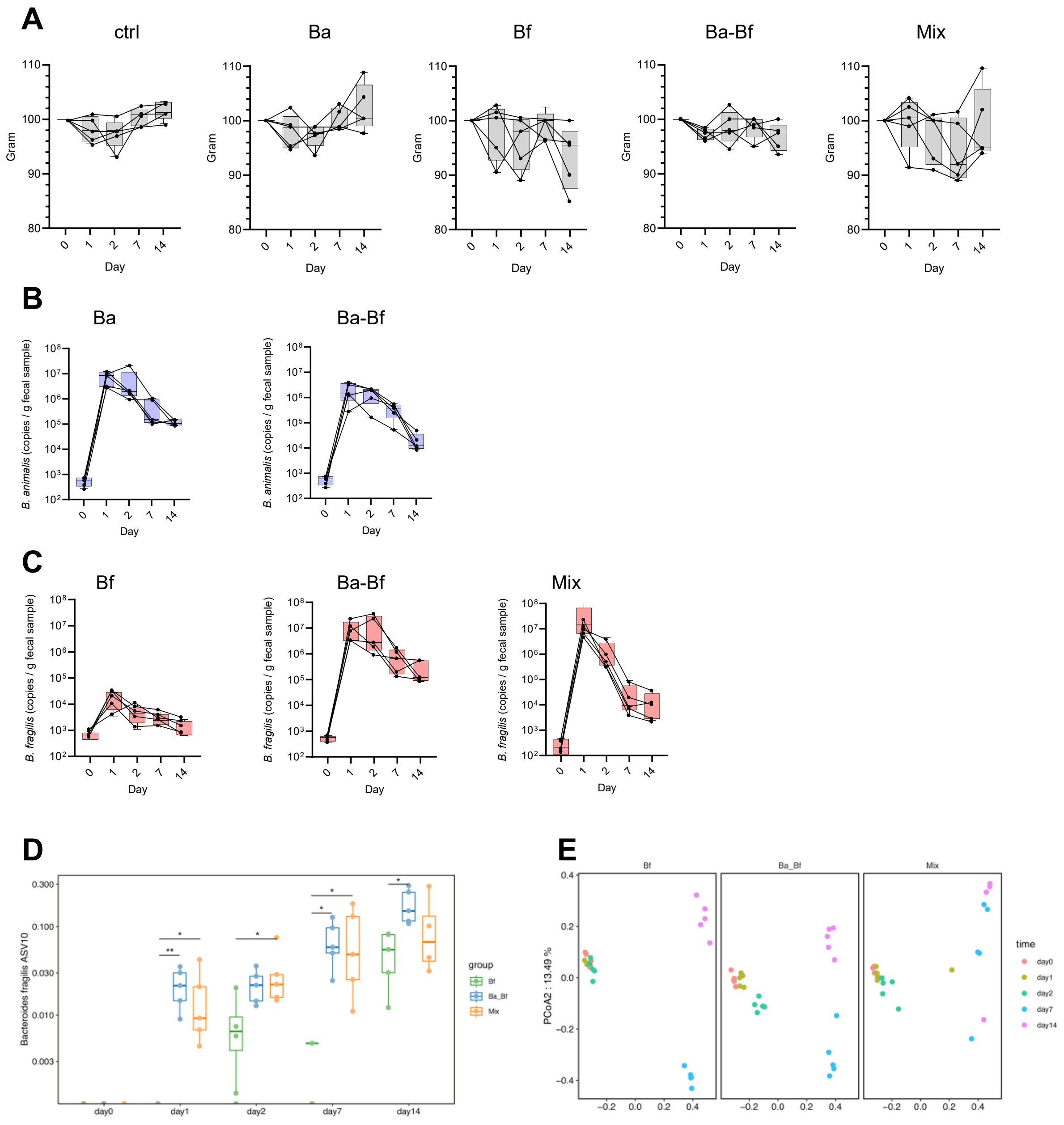

Fig S9. Related to **Fig 4**. Effects of bacterial treatments on mouse weight and colonization dynamics. (A) Body weight trajectories of AVNM-treated mice over 14 days following colonization with different bacterial groups: control (ctrl), *B. animalis* subsp. *lactis* (Ba), *B. fragilis* (Bf), co-colonization (Ba-Bf), and metabolite mixture followed by *B. fragilis* (Mix). All groups maintained relatively stable weight throughout the experiment, indicating good health and tolerance to treatments. (B) Fecal abundance of *B. animalis* subsp. *lactis* in Ba and Ba-Bf groups, measured by qPCR. Sustained colonization was observed over time. (C) Fecal abundance of *B. fragilis* in Bf, Ba-Bf, and Mix groups, showing enhanced colonization in the presence of Ba or its metabolites. Data are presented as mean ± SEM. (D) Relative abundance of *B. fragilis* (ASV10) in fecal samples collected at days 0, 1, 2, 7, and 14 (n = 5 per group). Data are shown as

box plots: the horizontal line indicates the median, the box represents the interquartile range (IQR), and whiskers extend to the minimum and maximum values. Each dot represents an individual mouse. Statistical significance was assessed using the Kruskal–Wallis test followed by Dunn's post-hoc test (*, $p < 0.05$ and **, $p < 0.01$). The Bf, Ba-Bf and Mix groups exhibited a time-dependent increase in *B. fragilis* abundance, with significant differences observed between groups at multiple time points. (E) Principal Coordinates Analysis (PCoA) of fecal microbiota composition across three treatment groups (Bf, Ba-Bf, Mix) over time. Samples were collected at days 0, 1, 2, 7, and 14 and analyzed using Bray–Curtis dissimilarity. Each colored dot represents an individual mouse at a specific time point. Temporal shifts in microbial community structure were observed within each group, with the Mix condition showing pronounced separation of day 7 and day 14 samples from earlier time points, indicating dynamic community restructuring.
